# Emu Oil and zinc monoglycerolate independently reduce disease severity in a rat model of ulcerative colitis

**DOI:** 10.1007/s10534-023-00521-w

**Published:** 2023-07-05

**Authors:** Suzanne Mashtoub, Gordon S Howarth

**Affiliations:** 1 Department of Gastroenterology, Women’s and Children’s Hospital, 72 King William Road, North Adelaide, SA 5006 Australia; 2 Discipline of Physiology, School of Biomedicine, The University of Adelaide, Adelaide, SA Australia; 3 School of Medicine, The University of Western Australia, Perth, WA Australia; 4 School of Animal and Veterinary Sciences, The University of Adelaide Roseworthy Campus, Roseworthy, SA Australia

**Keywords:** Zinc monoglycerolate, Emu Oil, Ulcerative colitis, Rats

## Abstract

Ulcerative colitis is characterized by colonic inflammation. Previously, Emu Oil protected the intestine against experimentally-induced inflammatory intestinal disorders. Zinc monoglycerolate (ZMG) polymer, formed by heating zinc oxide with glycerol, demonstrated anti-inflammatory and wound healing properties. We aimed to determine whether ZMG, alone or in combination with Emu Oil, could reduce acute colitis severity in rats. Male Sprague Dawley rats (n = 8/group) were orally-administered either vehicle, ZMG, Emu Oil (EO) or ZMG combined with EO (ZMG/EO) daily. Rats were provided *ad libitum* access to drinking water (Groups 1–4) or dextran sulphate sodium (DSS; 2%w/v; Groups 5–8) throughout the trial (days 0–5) before euthanasia on day 6. Disease activity index, crypt depth, degranulated mast cells (DMCs) and myeloperoxidase (MPO) activity were assessed. p < 0.05 was considered significant. DSS increased disease severity (days 3–6) compared to normal controls (p < 0.05). Importantly, in DSS-administered rats, ZMG/EO (day 3) and ZMG (day 6) reduced disease activity index compared to controls (p < 0.05). Following DSS consumption, distal colonic crypts lengthened (p < 0.01), occurring to a greater extent with EO compared to ZMG and ZMG/EO (p < 0.001). DSS increased colonic DMC numbers compared to normal controls (p < 0.001); an effect decreased only by EO (p < 0.05). Colonic MPO activity increased following DSS consumption (p < 0.05); notably, ZMG, EO and ZMG/EO treatments decreased MPO activity compared to DSS controls (p < 0.001). EO, ZMG and ZMG/EO did not impact any parameter in normal animals. Emu Oil and ZMG independently decreased selected indicators of colitic disease severity in rats; however, the combination did not reveal any additional benefit.

## Introduction

Ulcerative colitis is a chronic, idiopathic, relapsing and incurable intestinal inflammatory disorder, characterised by severe damage to the distal colonic mucosa (Hendrickson et al. 2002). Therapeutic options are variably effective; thus, new treatment approaches are required.

Zinc, an essential trace element, is required for wound healing processes via its anti-inflammatory, antioxidant and angiogenic actions, mast cell stabilisation, growth of granulation tissue and epithelium and inhibition of bacterial growth (Cagen and Klaassen 1979; Chasapis et al. 2012; Chvapil et al. 1973; Penissi et al. 2003). Zinc monoglycerolate (ZMG) is a lubricious, two-dimensional polymer formed by heating zinc oxide with glycerol. Unlike zinc oxide, ZMG is readily absorbed into tissues where it slowly releases Zn^2+^ ions (Rainsford and Whitehouse 1992). In a rat model of adjuvant-induced polyarthritis, zinc repletion by parenteral administration of ZMG suppressed chronic inflammation (Whitehouse et al. 1990). Additionally, orally-administered ZMG was more efficacious than zinc sulphate and other zinc salts or metal ion complexes in gastric ulcer models in mice (Rainsford and Whitehouse 1992). Randomised, placebo-controlled clinical trials of oral and circumoral herpes highlighted the efficacy of topically-applied ZMG (Apisariyakulm et al. 1990; Godfrey et al. 2001). Zinc is known to possess both mast cell stabilising and gastrointestinal cytoprotective activity (Penissi et al. 2003). A zinc-based product such as ZMG would therefore appear ideally suited for the treatment of inflammatory conditions including ulcerative colitis. As ZMG is poorly soluble in water (Fairlie et al. 1992; Whitehouse et al. 1990), the utilisation of a vehicle with known therapeutic benefit, such as Emu Oil, could theoretically facilitate ZMG uptake whilst exerting synergistic efficacy.

Emu Oil, extracted from emu adipose tissue, is highly permeable with its skin permeation properties being exploited in skin moisturizing products (Abimosleh et al. 2012b; Mashtoub 2017). Emu Oil has a lipid content of 98.0% (Abimosleh et al. 2012b) and variable levels of compounds with antioxidant properties (Chartier et al. 2019; Mashtoub 2017). Previously, anti-inflammatory and reparative properties of orally-administered Emu Oil have been described in animal models of acute (Abimosleh et al. 2012a) and chronic colitis (Safaeian et al. 2019), colitis-associated colorectal cancer (Chartier et al. 2018, 2021a, b; Mashtoub et al. 2022), acute Crohn’s-like colitis (Mitchell et al. 2020), non-steroidal anti-inflammatory drug-induced enteropathy (Abimosleh et al. 2013) and chemotherapy-induced intestinal mucositis (Mashtoub et al. 2013, 2016; Raghu Nadhanan et al. 2012).

The dextran sulphate sodium (DSS) model of experimental colitis is one of the most widely utilised animal models of inflammatory bowel disease (Tran et al. 2012). Oral consumption of DSS results in histopathological features resembling the damage manifest in human ulcerative colitis patients (preferentially-affecting the distal colon) and elicits an inflammatory response consistent with human inflammatory bowel disease. DSS administration also produces overt signs of ulcerative colitis disease activity including rectal bleeding, weight loss and diarrhea (Tran et al. 2012).

We sought to determine if independent or combined administration of ZMG and Emu Oil could decrease disease severity in a rat model of acute DSS-induced colitis.

## Methodology

### General experimental procedures

Male Sprague Dawley rats (8 weeks of age; 135–150 g; mean day 0 bodyweight 141 g) were sourced from Laboratory Animal Services, The University of Adelaide (Adelaide, South Australia, Australia) and housed at the Research Facility, Roseworthy Campus, The University of Adelaide (Roseworthy, South Australia, Australia). Rats were individually housed in metabolism cages (Tecniplast, West Chester, Pennsylvania, USA) at room temperature with a light:dark cycle of 12 h. All rats were provided *ad libitum* access to a standard 18% casein-based diet (Tomas et al. 1991) and water and were acclimatized for three days prior to experimentation. All animal studies were conducted in compliance with the ‘Australian code for the care and use of animals for scientific purposes 8th edition (2013)’ under approval from the Animal Ethics Committee of The University of Adelaide (approval number M-2015-053).

### Experimental groups

Rats (n = 64) were assigned to eight groups (n = 8/group) by random stratification based on initial bodyweight; Group 1: Water + Vehicle, Group 2: Water + ZMG, Group 3: Water + Emu Oil (EO), Group 4: Water + ZMG/EO, Group 5: DSS + Vehicle, Group 6: DSS + ZMG, Group 7: DSS + EO, Group 8: DSS + ZMG/EO.

### Test compounds

Emu Oil was prepared utilizing specific methodologies developed for Technology Investment Corporation by Emu Tracks (Marleston, South Australia, Australia). Briefly, these processes involved the rendering and filtration of Emu adipose tissue, with appropriate considerations for delivery of quality assurance and product consistency. ZMG (Story Pharmaceutics Pty Ltd, Adelaide, South Australia, Australia; 30 mg made up to 1 ml with water), Emu Oil (420 mg Emu Oil made up to 1 ml with water), ZMG/EO (15 mg ZMG + 210 mg Emu Oil made up to 1 ml with water), or vehicle (1 ml water) was administered daily (from days 0 to 5) via oral gavage using a blunted 18-gauge needle attached to polyethylene tubing. Groups 5–8 consumed 2% DSS (MW: 40,000; MP Biomedicals, Solon, Ohio, USA) *ad libitum* in drinking water throughout the experimental period to induce experimental colitis.

### Daily measurements

Body weight, food and water intake, and faecal (wet weight) and urine output were monitored and measured daily. Fluid balance was determined by the difference between fluid intake and urine output, expressed as mean daily change. The severity of colitis was assessed daily using a disease activity index based on four parameters scored from 0 to 3 (maximal severity): body weight loss, rectal bleeding, stool consistency and overall general condition, which was summed to achieve an overall disease activity index, as described previously (Howarth et al. 2000; Murthy et al. 1993).

### Tissue collection

On day 6, rats were euthanised by CO_2_ asphyxiation followed by cervical dislocation. Whole blood was collected via cardiac puncture for complete blood picture analyses (Veterinary Diagnostics Laboratory, The University of Adelaide), including counts of white blood cells, neutrophils (units and %), lymphocytes (units and %), monocytes (units and %), basophils (units and %), eosinophils (units and %), haematocrit, haemoglobin, mean cell haemoglobin, mean cell haemoglobin concentration, mean cell volume, red blood cells, red cell distribution width, platelets and mean platelet volume.

Visceral organs, including heart, liver, spleen, thymus, lungs and left and right kidneys and caecum were weighed and discarded. Weights and lengths of the duodenum, small intestine (jejunum-ileum) and colon were recorded. Segments of colon (2 cm; proximal and distal) were removed and placed in 10% buffered formalin for histological analyses. Additionally, 4 cm mid-colonic segments (directly adjacent to the corresponding histological samples) were collected, snap-frozen in liquid nitrogen and stored at − 80 °C for biochemical analysis.

### Histological analyses

Proximal and distal colon samples were routinely processed and embedded in paraffin wax. Sections (4 μm) were then stained with haematoxylin and eosin (H&E). Measurements of crypt depth in the proximal and distal colon were determined for 40 well-orientated crypts per tissue section per rat and a mean value was then obtained (Abimosleh et al. 2012a). All analyses were performed in a blinded fashion, using an Olympus BH-2 light microscope (Olympus Corporation, Tokyo, Japan) and Olympus Soft Imaging Solutions GmbH software analysis version 5.2 (Tokyo, Japan).

For each rat, multiple transverse sections were selected from colonic samples (average four sections reviewed per sample) from H&E-stained slides (slide 1) and Toluidine blue (slide 2). Control tissue for Toluidine blue staining was a canine mast cell tumour and controls were performed with each batch of stained sections. Degranulated mast cell (DMC) and mast cell (MC) counts were performed manually using a light microscope and MC and DMCs distinguished on basis of morphology in Toluidine blue stained slides (DMCs retain few stained granules via Toluidine blue and have distinctive nuclear and cytoplasmic morphology to enable accurate detection). DMC and MC were enumerated within the lamina propria (above muscularis mucosae) and then enumerated in the submucosa/ muscularis/ serosae per high power (400 ×; 0.238 mm^2^) field, in 10 non-contiguous fields per colon sample. Average colonic DMCs and MCs were determined per 0.238 mm^2^ field.

### Biochemical analysis

Myeloperoxidase levels in the colon were determined as an indicator of neutrophil infiltration, and hence, acute inflammation, using techniques described by Howarth et al. (Howarth et al. 1996). Thawed, homogenised 4 cm samples of colon were centrifuged at 13,000 g for 12 min, after which the supernatant was discarded, and the tissue homogenate was re-suspended in 200 µL of 0.5% hexadecyltrimethyl ammonium bromide buffer, a detergent (Sigma Chemicals, Castle Hill, NSW, Australia). Samples were vortexed for 2 min and further centrifuged at 13,000 g for 2 min. Background, negative and positive control samples (50 µL) and the supernatants of each test sample were then aliquoted into duplicate wells of a microtitre 96-well plate. Following the addition of a reaction solution (200 µL to each well; 4.2 mg of O-dianisidine dihydrochloride reagent, 12.5 µL H_2_O_2_, 2.5 ml potassium phosphate buffer [pH 6.0], 22.5 mL distilled water) the change in absorbance was measured at 450 nm at one minute intervals for 15 min with a spectrophotometer (Victor X4 Multilabel Reader, Perkin Elmer, Singapore). Data were expressed as MPO units per gram of tissue.

### Statistical analysis

Statistical comparisons were performed using SPSS version 22.0 for Windows (SPSS Inc. Chicago, Illinois, USA). Data were tested for normality using a Shapiro–Wilk test. Daily metabolic data were analyzed using repeated measures ANOVA with least significance difference (LSD) to compare the differences both among and within groups. Tissue analyses were analyzed using a one-way ANOVA, with a Tukey’s post hoc test. All parametric data were expressed as mean ± standard error of the mean (SEM). Disease activity index comparisons between groups each day were made using a Kruskal Wallis test with Mann–Whitney U tests and expressed as median (range). For all analyses, p < 0.05 was considered significant.

## Results

### Daily metabolic data

#### Bodyweight

**Fig. 1:**
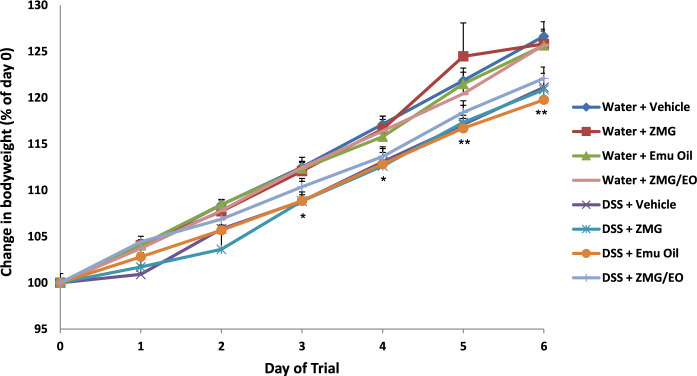
Daily body weight change. Data are expressed as mean (body weight change from day 0 starting bodyweight; %) ± standard error of the mean. *p < 0.05, **p < 0.01 compared to Water + Vehicle

Bodyweight was determined as a mean percentage change from starting bodyweight (100% on day 0), represented as daily **(**Fig. 1**)** and total bodyweight change (from day 0–6; data not shown). Throughout the experimental period, there were no significant changes in daily bodyweight gain following ZMG, Emu Oil or the combination of ZMG and Emu Oil (ZMG/EO) in normal rats, compared to controls (Fig. 1). Between days 3–6, daily bodyweight gain was significantly decreased in DSS-treated rats, compared to vehicle controls (p < 0.05; maximum 5.5% lower on day 6). Amongst DSS-treated groups, ZMG, Emu Oil and ZMG/EO failed to significantly affect bodyweight gain during these time points. Similarly, total bodyweight change remained consistent for all treatment groups (p > 0.05; data not shown).

#### Daily fluid intake

**Fig. 2:**
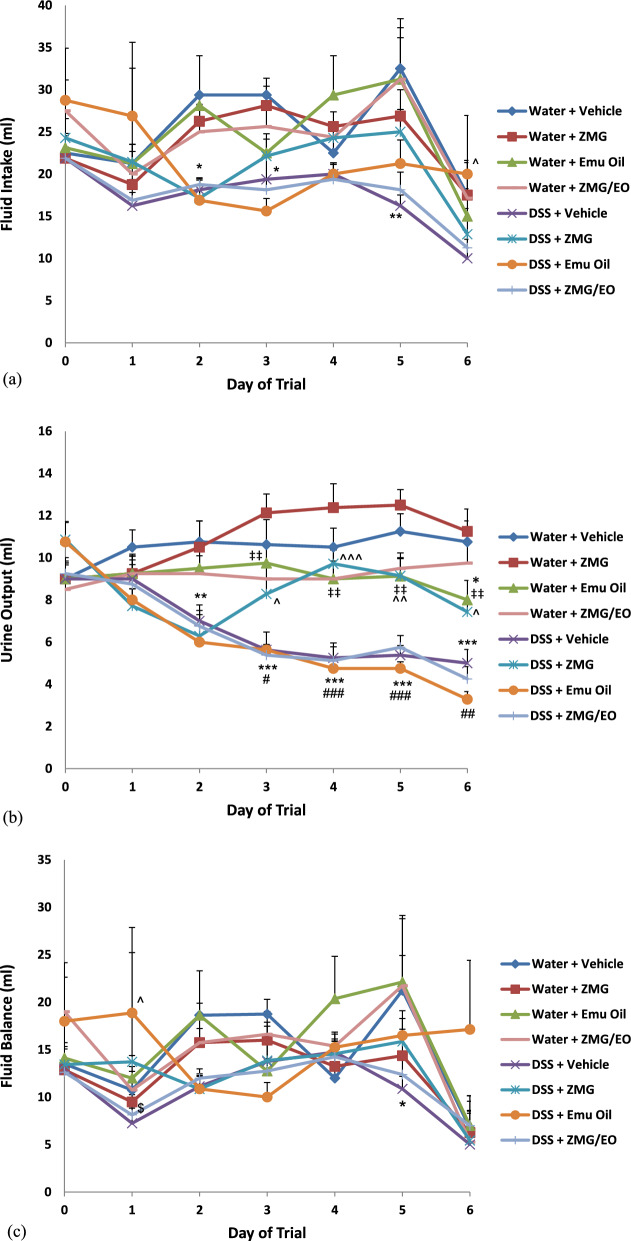
Daily **a** fluid intake, **b** urine output and **c** fluid balance. Data are expressed as mean (ml) ± standard error of the mean. *p < 0.05, **p < 0.01, ***p < 0.001 compared to Water + Vehicle; ‡‡p < 0.01 compared to Water + ZMG; ^p < 0.05, ^^p < 0.01, ^^^p < 0.001 compared to DSS + Vehicle; #p < 0.05, ##p < 0.01, ###p < 0.001 compared to DSS + ZMG; $p < 0.05 compared to DSS + Emu Oil

In normal animals, daily fluid (water) intake remained unchanged following ZMG, Emu Oil or ZMG/EO treatment, compared to controls (p > 0.05; Fig. 2a). However, during days 2, 3 and 5, DSS-control rats consumed significantly less fluid compared to normal vehicle controls (p < 0.05; maximum 50% reduction on day 5). In DSS-treated animals, Emu Oil significantly increased DSS fluid intake on day 6, compared to water-drinking controls (p < 0.05; Fig. 2a).

#### Daily urine output

Compared to normal controls, daily urine output was not significantly different following any treatment **(**Fig. 2b**)**. However, in normal animals, Emu Oil treatment resulted in significantly reduced urine output compared to animals treated with ZMG (days 3–6; p < 0.01). DSS consumption resulted in significantly reduced daily urine output compared to normal vehicle controls on days 2–6 (p < 0.01; maximum 53.5% urine reduction on day 6). Moreover, in DSS-treated animals, ZMG administration resulted in significantly increased daily urine output on days 3–6 compared to both DSS controls and Emu Oil-treated animals (Fig. 2b; p < 0.05).

#### Daily fluid balance

On day 5, fluid balance was significantly decreased in DSS control animals compared to normal controls (p < 0.05; Fig. 2c). In DSS-treated animals, Emu Oil resulted in significantly elevated fluid balance compared to both controls and animals treated with ZMG/EO (day 1; Fig. 2c; p < 0.05). There were no significant differences amongst groups on all other days (p > 0.05).

#### Daily food intake

**Fig. 3:**
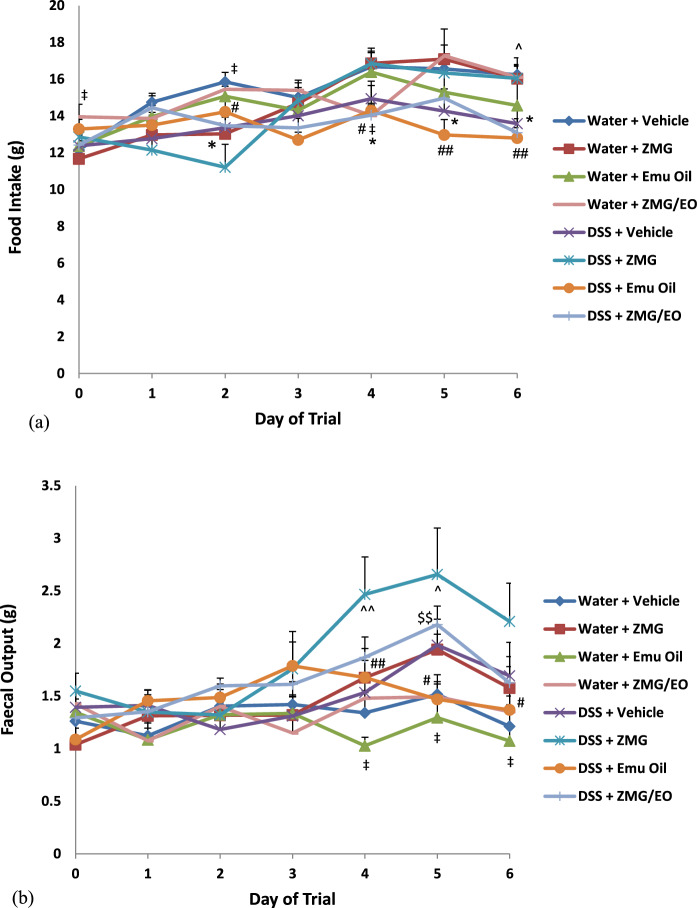
Daily **a** food intake and **b** faecal output. Data are expressed as mean (g) ± standard error of the mean. *p < 0.05 compared to Water + Vehicle; ‡p < 0.05 compared to Water + ZMG; ^p < 0.05, ^^p < 0.01 compared to DSS + Vehicle; #p < 0.05, ##p < 0.01 compared to DSS + ZMG; $$p < 0.01 compared to DSS + Emu Oil

In normal animals, ZMG decreased food intake compared to normal controls on day 2 and compared to ZMG/EO-treated rats on day 1 (Fig. 3a; p < 0.05). ZMG/EO administration in normal animals decreased food intake on day 4, compared to normal controls (p < 0.05). DSS control animals consumed less food on days 4–6 compared to normal controls (p < 0.05; maximum 16% food intake reduction on day 6). In DSS-treated rats, ZMG administration increased food intake on day 6 compared to controls (18%) and on days 4–6 compared to Emu Oil-treated rats (Fig. 3a; p < 0.05).

#### Daily faecal output

Daily faecal output (wet weight) was significantly reduced in normal animals treated with Emu Oil, compared to ZMG-treated animals on days 4–6 (Fig. 3b; p < 0.05). All other normal animal groups remained unchanged throughout the trial (p > 0.05). Furthermore, there were no significant differences amongst normal controls and DSS controls during the experimental period. In DSS-treated animals, ZMG increased daily faecal output on days 4 (61%) and 5 (34%) compared to controls; and on days 4–6 compared to Emu Oil treatment (maximum 60% faecal output increase on day 5) (Fig. 3b; p < 0.05).

### Disease activity index

ZMG, Emu Oil and ZMG/EO did not impact the disease activity index in normal animals, with no significant differences in disease severity among normal treatment groups **(**Table 1**)**. However, DSS consumption significantly increased disease severity on days 3–6 compared to normal controls (median 0; range 0), reaching a peak disease activity index score of median 3 (range 5) on day 6 (Table 1; p < 0.001); disease activity was primarily driven by increased stool consistency (data not shown). Importantly, in rats ingesting DSS, ZMG in combination with Emu Oil (day 3) and ZMG treatment (day 6) significantly reduced disease activity compared to control rats (Table 1; p < 0.05).

**Table 1:** Disease activity index based on four parameters including body weight loss, rectal bleeding, stool consistency and overall general condition

	Water				DSS			
	Vehicle	ZMG	Emu Oil	ZMG+Emu Oil	Vehicle	ZMG	Emu Oil	ZMG+Emu Oil
Day 0	0 (0)	0 (1)	0 (1)	0 (0)	0 (0)	0 (0)	0 (0)	0 (1)
Day 1	0 (0)	0 (0)	0 (0)	0 (0)	0 (1)	0 (1)	0 (0)	0 (0)
Day 2	0 (0)	0 (0)	0 (0)	0 (0)	0 (0)	0 (3)	0 (1)	0 (1)
Day 3	0 (0)	0 (1)	0 (0)	0 (0)	1 (1)***	1 (1)	0.5 (1)	0 (1)^
Day 4	0 (0)	0 (0)	0 (1)	0 (0)	1 (2)*	1 (1)	1 (2)	1 (2)
Day 5	0 (0)	0 (0)	0 (0)	0 (1)	1 (3)***	1 (2)	1 (4)	1 (3)
Day 6	0 (0)	0 (1)	0 (1)	0 (0)	3 (5)***	2 (3)^	3.5 (7)	2 (6)

Data are expressed as median (range). *p 0.05, ***p 0.001 compared to Water + Vehicle; ^p 0.05 compared to DSS + Vehicle

### Tissue data

#### Visceral organ weights

No significant differences amongst treatment groups (data not shown) were evident in any visceral organ weights (heart, liver, spleen, thymus, lungs and left and right kidneys), expressed as a proportion of bodyweight (%).

#### Intestinal organ weights

**Fig. 4:**
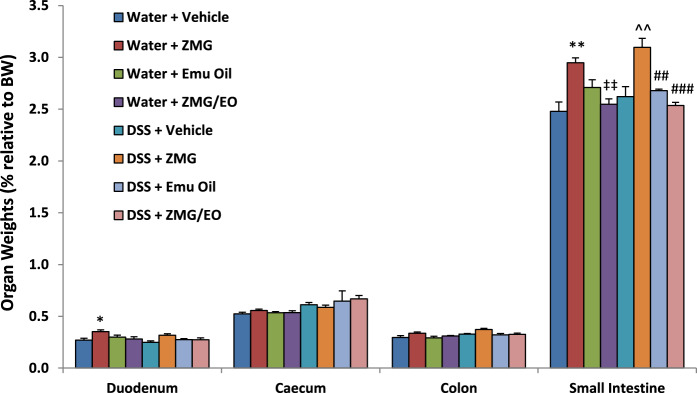
Intestinal organ weights following body weight adjustment. Small intestine: proximal jejunum to distal ileum. Data are expressed as mean (% relative to body weight; BW) ± standard error of the mean. **p < 0.01 compared to Water + Vehicle; ‡‡p < 0.01 compared to Water + ZMG; ^^p < 0.01 compared to DSS + Vehicle; ##p < 0.01; ###p < 0.001 compared to DSS + ZMG

In normal animals, duodenal weights were significantly greater in ZMG-treated rats, compared to controls (Fig. 4; p < 0.05). However, duodenal weights (expressed as % relative to bodyweight) in all other groups remained unchanged (p > 0.05); there were no differences in duodenal weight per cm among groups (data not shown; p > 0.05). Moreover, ZMG administration in normal animals resulted in increased small intestinal weights compared to normal vehicle controls (19%) and compared to ZMG/EO-treated normal animals (Fig. 4; p < 0.01). Similarly, in DSS-treated groups, ZMG administration significantly increased small intestinal weights compared to DSS controls (18%) and Emu Oil and ZMG/EO groups (Fig. 4; p < 0.01). Small intestinal weight per cm remained unchanged across all treatments (p > 0.05; data not shown). Caecum weights were not significantly different between any of the treatment groups (Fig. 4). Colon weights did not differ among treatment groups when represented as % relative to bodyweight (Fig. 4); however, colon weight per cm was significantly greater in DSS control rats (0.09 ± 0.01) compared to healthy controls (0.06 ± 0.00; p < 0.001), with no treatment effect (data not shown; p > 0.05).

#### Organ lengths

**Fig. 5:**
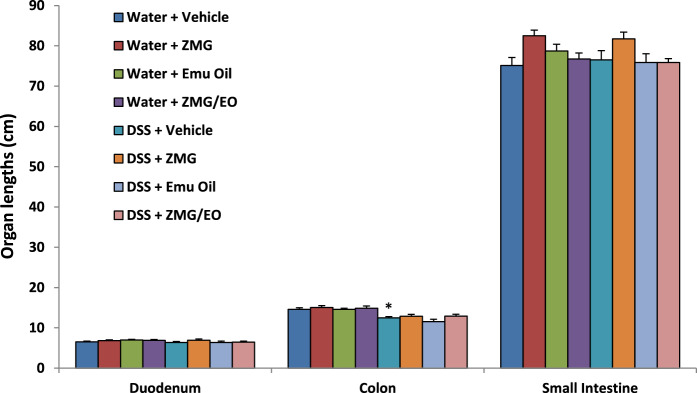
Intestinal organ lengths. Data are expressed as mean (cm) ± standard error of the mean. *p < 0.05 compared to Water + Vehicle

Colon lengths were significantly decreased in DSS control animals compared to normal controls (p < 0.05), which was not improved by any treatment **(**Fig. 5**)**. Duodenal and small intestinal lengths were not significantly impacted by any treatment (p > 0.05).

### Blood analytes

**Fig. 6:**
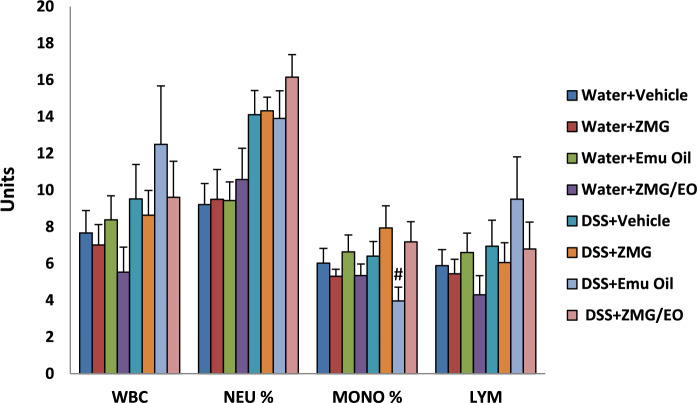
Blood analytes including white blood cells (WBC); neutrophils % (NEU%); monocytes % (MONO%); lymphocytes (LYM). Data are expressed as mean (units) ± standard error of the mean. #p < 0.05 compared to DSS + ZMG.

In DSS-treated animals, the red cell distribution width (RDW units) was significantly lower in ZMG/EO-treated animals (14.49 ± 0.24) compared to ZMG treatment (16.21 ± 0.52; p < 0.05), with no differences evident between other treatment groups (data not shown; p > 0.05). Furthermore, monocytes (%) were significantly reduced in Emu Oil-treated rats consuming DSS (3.97 ± 0.75), compared to those treated with ZMG (7.94 ± 1.2; p < 0.05), with no differences among other treatment groups (Fig. 6). No significant differences were evident in any other blood analytes amongst treatment groups, including counts for white blood cells, neutrophils (%), lymphocytes (units) (Fig. 6), neutrophils (units), lymphocytes (%), monocytes (units), basophils (units and %) and eosinophils (units and %). Similarly there were no significant differences for haematocrit, haemoglobin, mean cell haemoglobin, mean cell haemoglobin concentration, mean cell volume, red blood cells, red cell distribution width, platelets and mean platelet volume (data not shown).

### Crypt depth

**Fig. 7:**
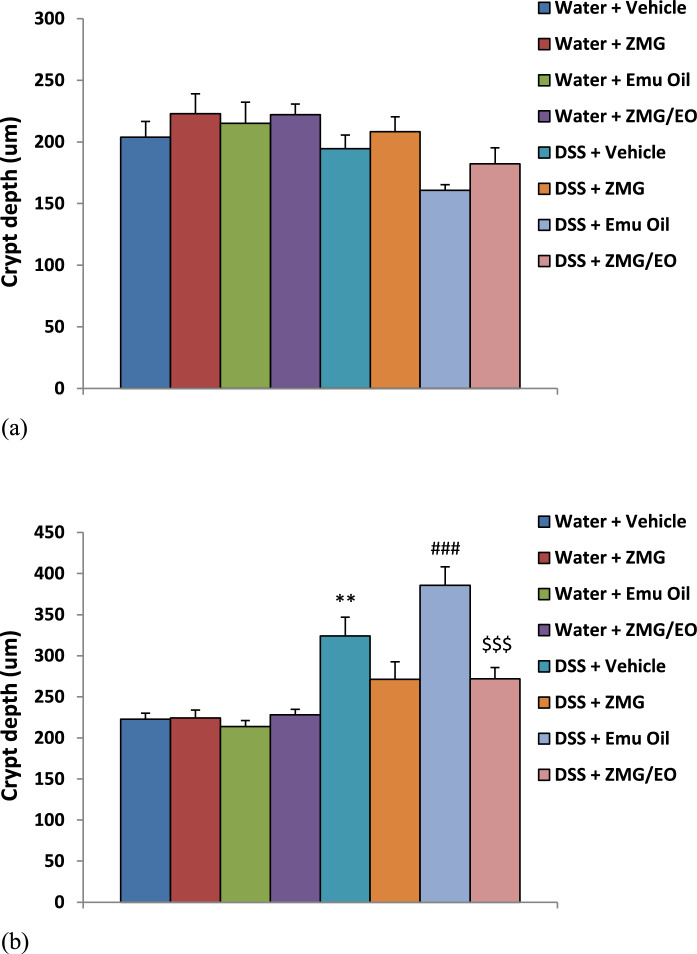
(a) Proximal colon and (b) distal colon crypt depth measurements. Data are expressed as mean (crypt depth; µm) ± standard error of the mean. **p < 0.01 compared to Water + Vehicle; ###p < 0.001 compared to DSS + ZMG; $$$p < 0.001 compared to DSS + Emu Oil

In the proximal colon, crypt depth did not significantly vary amongst treatment groups **(**Fig. 7a**)**. In the distal colon, treatments did not significantly impact normal animals. However, following DSS consumption in untreated animals, crypts were significantly lengthened (45% greater; Fig. 7b; p < 0.01). Amongst DSS groups, Emu Oil, ZMG and ZMG/EO treatment did not affect crypt depth compared to controls (p > 0.05); however, crypt lengthening occurred to a greater extent with Emu Oil compared to ZMG and ZMG/EO treatment (42% greater; Fig. 7b; p < 0.001).

### Mast cells

**Fig. 8:**
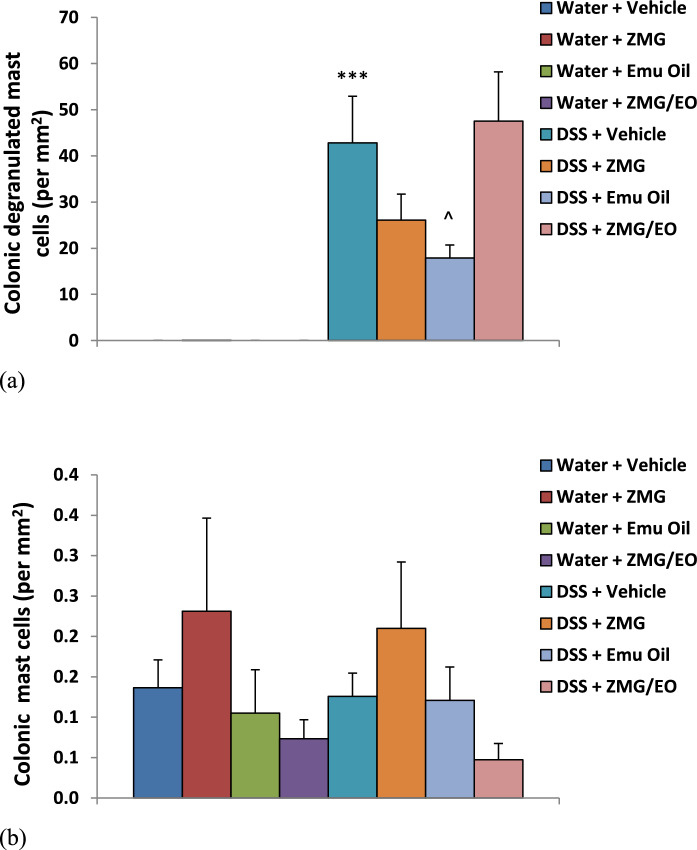
Numbers of colonic **a** de-granulated mast cells and **b** mast cells. Data are expressed as mean (count per mm^2^) ± standard error of the mean. ***p < 0.001 compared to Water + Vehicle; ^p < 0.05 compared to DSS + Vehicle

In normal animals, numbers of colonic DMCs were not significantly affected by any treatment **(**Fig. 8a**)**. However, following DSS consumption, colonic DMC numbers were significantly increased compared to normal controls (p < 0.001). In DSS-treated animals, Emu Oil significantly decreased DMC numbers compared to DSS-controls (58% reduction; Fig. 8a; p < 0.05). Although mean DMC number for ZMG treatment was lower than the DSS control, this failed to achieve statistical significance (p = 0.415). There was no significant impact following administration of ZMG/EO (Fig. 8a). Colonic MC numbers did not vary significantly amongst treatment groups **(**Fig. 8b**)**.

### Myeloperoxidase levels

**Fig. 9:**
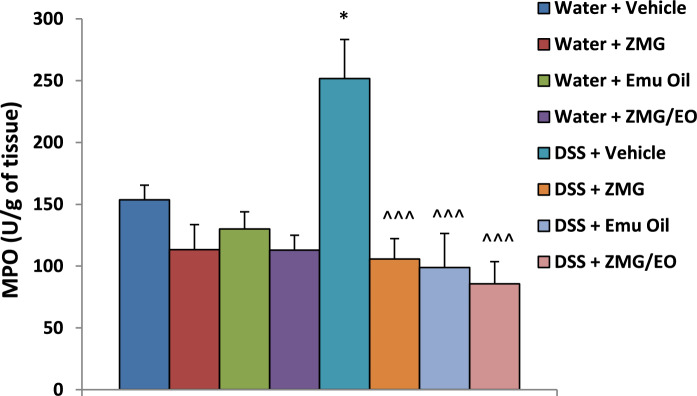
Colonic myeloperoxidase (MPO) activity (indicative of acute inflammation). Data are expressed as mean (MPO; units per gram of tissue) ± standard error of the mean. *p < 0.05 compared to Water + Vehicle; ^^^p < 0.001 compared to DSS + Vehicle

Myeloperoxidase (MPO) activity, indicative of acute inflammation, remained unchanged following any treatment in all normal animals (Fig. 9; p > 0.05). However, DSS consumption resulted in significantly elevated MPO activity compared to normal vehicle controls (64% greater; p < 0.05). Importantly, amongst DSS-treated groups, ZMG, Emu Oil and ZMG/EO treatments significantly decreased MPO activity compared to DSS controls (58%, 60.5% and 66% less MPO activity respectively; Fig. 9; p < 0.001).

## Discussion

ZMG represents an ideal pro-drug for the slow release of zinc ions into the intestinal mucosa. Although adequate zinc ingestion is associated with optimised intestinal digestive function, immune status and barrier function, effects on intestinal growth have generally not been well-described. In the current study, ZMG increased duodenal and small intestinal weights in both normal healthy rats and rats with induced colitis. Similar effects are often evident following administration of peptide growth factors such as epidermal growth factor (Zhao et al. 2002), insulin-like growth factor 1 (Howarth 2003; Howarth and Shoubridge 2001; Howarth et al. 1998; Howarth et al. 2000) and glucagon-like peptide 2 (Geier et al. 2005; Yazbeck et al. 2010a, b). The current findings could therefore be indicative of a potential intestino-trophic mechanism for ZMG. Previously, zinc has been demonstrated to partially alleviate features of small intestinal mucositis (Musa et al. 2015; Tran et al. 2009); however, concurrent improvements in large bowel functioning have not yet been described. Although a preliminary finding, the current study could represent a new mode of action for ZMG, suggesting potential application for disorders of the small intestine in which fortification of the intestinal mucosa is desirable. These include intestinal mucositis, the infective enteropathies, NSAID-enteropathy, small intestinal Crohn’s disease and short bowel syndrome.

Zinc and its binding protein metallothionein have been proposed to suppress disease activity in ulcerative colitis by sequestering free radicals (Tran et al. 2007). To this end, Iwaya et al. (2011) reported an exacerbation of DSS-colitis in rats via marginal zinc deficiency (Iwaya et al. 2011) whilst Tran et al. (2007) described a 50% reduction in disease activity following administration of zinc oxide in mice with DSS-colitis (Tran et al. 2007). On the final day of the current study (day 6), ZMG reduced clinically-assessed colitic disease activity and improved food intake with an associated increase in faecal output. This was accompanied by decreased colonic myeloperoxidase activity, representing a reduction in acute colonic inflammation. Considered together with the ZMG-induced increase in small intestinal weight, these findings indicate the therapeutic potential of ZMG for the coincident optimisation of small intestinal functioning and the control of colonic inflammation in colitis sufferers.

Emu Oil has been reported to stimulate repair of the colon in DSS-colitis in rats, as evidenced by crypt lengthening (Abimosleh et al. 2012a; Safaeian et al. 2019). Abimosleh et al. (2012a) demonstrated that prophylactic and therapeutic treatment with orally-administered Emu Oil (0.5 ml and 1 ml daily) in colitic rats significantly lengthened proximal and distal colonic crypts; this occurred to a greater extent than in colitic controls, indicating an enhanced recovery rate. Moreover, in a mouse model of DSS-induced chronic colitis (51 days), thrice weekly oral administration of Emu Oil (160 µl) reduced clinically-assessed disease severity, particularly evident during peak disease activity (days 0–6 and 21–27). The current study reflected enhanced compensatory crypt lengthening of the distal colon, complemented by a reduction in myeloperoxidase activity. Moreover, decreased numbers of degranulated mast cells following Emu Oil treatment may represent a key mechanism of therapeutic efficacy in a variety of gastrointestinal conditions. Parisio et al. (2020) reported a similar reduction in numbers of degranulated mast cells following oral administration of extra virgin olive oil in a rat model of dinitrobenzenesulfonic acid-induced colitis; an effect that was attributed to the phenolic compounds of the olive, particularly oleuropein and hydroxytyrosol (Parisio et al. 2020). Although a definitive mechanism of action for Emu Oil remains undefined, it is proposed that the ratio of n-6:n-9 fatty acids together with the non-triglyceride constituents (carotenoids, flavones, polyphenols, tocopherols) could play an important role (Mashtoub et al. 2014). Nevertheless, in the current study, the indications of clinical efficacy for Emu Oil against acute colitis were relatively minor at the dose and frequency of administration tested.

The combination of Emu Oil and ZMG did not confer any additional therapeutic benefit in the experimental setting of DSS-colitis when compared to the independent administration of Emu Oil and ZMG, with no indications of additive or synergistic efficacy. Zinc salts have been explored widely for both protection and treatment of intestinal inflammation when combined with a wide range of biologically active compounds. These have included probiotics (Lima et al. 2014; Musa et al. 2015; Park et al. 2018; Scrimgeour and Condlin 2009) and whey-derived growth factors (Tran et al. 2003). The latter study in particular demonstrated increased efficacy of the zinc/whey growth factor combination when used for the treatment of experimental mucositis (small intestinal condition), however, similar studies have not yet been conducted in ulcerative colitis.

Dissociation or absorption characteristics of zinc from ZMG in the upper intestinal tract should be explored. Findings may provide a potential mode of action for ZMG (topical versus systemic effect) in distal intestinal conditions. To date, Emu Oil in combination with other agents has not been widely investigated in the setting of gastrointestinal disease (Chartier et al. 2019). Mashtoub et al. (2016) reported a partial amelioration of chemotherapy-induced intestinal mucositis following treatment with Emu Oil in combination with a New Zealand Green-Lipped Mussel extract (Mashtoub et al. 2016). In 2021, Chartier et al. highlighted clinical efficacy and a reduction in numbers of colonic tumours following treatment with Emu Oil in combination with grape seed extract (Chartier et al. 2021b) and Saireito (a traditional Japanese medicine) (Chartier et al. 2021a) in mouse models of colitis-associated colorectal cancer. The current study therefore represents a similar novel approach investigating Emu Oil in combination with another bioactive compound in the context of bowel disease.

In conclusion, independent oral administration of ZMG and Emu Oil each resulted in indications of reduced disease activity in a rat model of acute colitis, although the combination of the two compounds failed to confer any additional benefit. Future studies of ZMG and Emu Oil in combination should investigate the impact of dose, frequency of administration and the proportions of the individual constituents on clinical efficacy.
